# The Doctrine of Reflexes

**Published:** 1892-07

**Authors:** 


### HOMŒOPATHIC PHYSICIAN,

A MONTHLY JOURNAL OF

HOMŒOPATHIC MATERIA MEDICA AND CLINICAL MEDICINE.

"If our school ever gives up the strict inductive method of Hahnemann, we are lost, and deserve only to be mentioned as a caricature in the history of medicine."—CONSTANTINE HERING.

Vol. XII. JULY, 1892. No. 7.

### EDITORIAL.

THE DOCTRINE OF REFLEXES.—The medical world is at present in a most remarkable state of mental disturbance by reason of the new doctrines as to the nature and cause of disease.

One of these doctrines, the germ theory of disease, is now in the zenith of its popularity. But it is being closely pressed by another one which is attracting an immense amount of attention and which the writer of this article heard the editor of *The Medical Investigator* predict would sooner or later supersede the germ theory.

This latest theory is the Doctrine of Reflexes; or, the principle that when a terminal of a given nerve is irritated in a particular manner, the impression made is transmitted backward to the junction of one of the branches of that nerve along which it is reflected to its terminals, where it gives expression to the irritation by the production of some other pathological phenomenon of a different and unexpected character and at a considerable distance from the seat of original irritation. "Man is a bundle of reflexes," writes one distinguished teacher of this doctrine.

One of the most familiar instances of this reflex principle is the toothache which occurs in a sound tooth at a distance from the seat of irritation which may be either a decay in another tooth or a dental abscess at its root.

Similarly we have "ciliary neuralgia" or headache as a reflex from eye-strain caused by defective accommodation of the crystalline lens.

Cases of epilepsy are reported as reflexes due to eye-strain; and the fitting of the eye with suitable glasses has caused the total disappearance of the convulsions.

Other cases of epilepsy are similarly reported which are reflexes caused by adherent foreskin to the glans penis. The operation of circumcision has completely relieved the patient. A case of this kind is reported in the *North American Practitioner* for May, page 207.

In the *Annals of Ophthalmology and Otology* for April, at page 138, is reported a remarkable case of coryza or common cold in the head due to eye-strain or defective vision. The case is thus described: "In the *Medical News* for March 12th, 1892, Dr. George M. Gould relates the striking case of a man forty-six years of age who had suffered every year, the entire winter through for eighteen years with severe cold, and who has enjoyed immunity from the distressing condition during the past two years in which he has had his refractive error corrected" by suitable lenses. Stronger lenses were, however, given that better distant vision might be obtained, but "the results were anything but satisfactory; for he caught cold the first day his stronger lenses were worn. During the three months following, the experiment of wearing the stronger lenses was repeated many times, but it was found that an hour's use always caused a return of his old-time trouble of the nose and throat, consisting of coryza, pharyngeal congestion, changed timbre of voice, etc. These symptoms always disappeared in a few hours upon a change to weaker lenses."

In the same journal, at page 106, Dr. Carl Seiler speaks of neuralgic headaches which are reflexes due to diseases of the nose such as nasal polypi, deviation of septum or hypertrophy of tissue covering the middle turbinated bone. "It is a wellknown fact," says Dr. Seiler, "that pressure upon the sensory branches of the nasal branch emanating from Meckles' ganglion, will produce pain of a neuralgic character in the other branches emanating from the same nerve centre, and thus frontal headaches and orbital neuralgias are so very commonly caused by pressure."

One of the logical results of this doctrine of reflexes is the practice of Orificial Surgery, which has made a sensation in the homœopathic school almost as great as Koch's method of treating tuberculosis has in the old school. According to the orificial philosophy nearly every ailment of the human system is a reflex caused by diseased conditions of the orifices of the body, notably the rectum. Treat these orifices and you cure the other ailments. This treatment in the case of the rectum consists principally of dilating or stretching the sphincter muscles guarding this orifice. A bivalve speculum collapsed is introduced into the rectum and then expanded and withdrawn. Thus the sphincter muscle is violently expanded. The process is indeed a species of nerve stretching, as it is apparent that the terminal filaments of the nerves in this locality are extended to the utmost by this means. Says Dr. L. G. Van Scoyoc, in *The Medical Standard* for April, 1892, in speaking of this method, "we do know that by the shocking of the nerves in the region of the lower orifices the circulation and respiration are affected as in no other way. This is shown by the stertorous respiration and the warming of the extremities during dilatation of the sphincters, or otherwise shocking or impinging upon these nerves in or about these orifices. Another and perhaps the strongest proof is that by relieving the improper tensions of the sphincters and removing all sources of irritation at these orifices, about four-fifths of all chronic diseases are cured or are made curable.

"Again it is a noticeable fact that disciples of the Orificial Philosophy have been on the alert and are still looking for a case of chronic disease in which no irritation exists at one or all the orifices named."

Dr. Pratt, the distinguished author of Orificial Surgery, declares that the relief obtained by its means " beggars belief." It is therefore the great panacea for everything. Accordingly we find reported by Dr. J. C. Daily, of Fort Smith, Arkansas, in *The Southern Journal of Homœopathy* for May, 1892, page 604, a case of Morphine poisoning, fifty-seven grains having been taken, in which " resuscitation was accomplished by stretching the sphincter ani with a bivalve speculum. "

Many more cases might be quoted in illustration of the principle of reflexes, but this article is already too long. Enough, however, have been furnished to give the reader a medical horizon and enable him to see that each specialist in medicine considers nearly every sick condition in patients as a reflex caused by irritation in the particular organ which it is in his province to treat. Remove the irritation and the reflex ailment disappears. Thus the sufferer must go about from one specialist to another until he has met the one who strikes the particular irritation of which his complaint is the reflex.

In this scheme the idea of selecting a curative agent by the laws of the similars is completely set aside, and no allusion made to it except by Dr. L. G. Van Scoyoc, before quoted, who declares in *The Argonaut* for May, page 74, that in the treatment of hemorrhoids he uses the indicated homœopathic remedy, and that if orificial surgery is resorted to, it is with the intention of " at least laying the foundation of cure for some chronic disease that may have baffled the skill of the best therapeutics for years."

When this new theory has been before the profession a sufficient length of time for experience to accumulate as to its actual value, it will be found to be not as efficacious as at first supposed. Failures will accumulate and disasters of different kinds will happen just as is the case with other modes of forcible interference with disease processes. The homœopathist therefore should not contemplate the abandonment of his materia medica nor allow his attention to be diverted from the study of it, because of this new doctrine which now seems to carry all before it.

